# Ribavirin Protects Syrian Hamsters against Lethal Hantavirus Pulmonary Syndrome — After Intranasal Exposure to Andes Virus

**DOI:** 10.3390/v5112704

**Published:** 2013-11-08

**Authors:** Monica Ogg, Colleen B. Jonsson, Jeremy V. Camp, Jay W. Hooper

**Affiliations:** 1 Molecular Virology Branch, United States Army Medical Research Institute of Infectious Diseases, Fort Detrick, MD 21772, USA; E-Mail: Monica.m.ogg.ctr@mail.mil; 2 Department of Microbiology and Immunology, Center for Predictive Medicine for Infectious Diseases and Biodefense, Louisville, KY 40202, USA; E-Mail: jvcamp01@exchange.louisville.edu

**Keywords:** hantaviruses, ribavirin, hamster, prophylactic, post-exposure antiviral

## Abstract

Andes virus, ANDV, harbored by wild rodents, causes the highly lethal hantavirus pulmonary syndrome (HPS) upon transmission to humans resulting in death in 30% to 50% of the cases. As there is no treatment for this disease, we systematically tested the efficacy of ribavirin *in vitro* and in an animal model. *In vitro* assays confirmed antiviral activity and determined that the most effective doses were 40 µg/mL and above. We tested three different concentrations of ribavirin for their capability to prevent HPS in the ANDV hamster model following an intranasal challenge. While the highest level of ribavirin (200 mg/kg) was toxic to the hamster, both the middle (100 mg/kg) and the lowest concentration (50 mg/kg) prevented HPS in hamsters without toxicity. Specifically, 8 of 8 hamsters survived intranasal challenge for both of those groups whereas 7 of 8 PBS control-treated animals developed lethal HPS. Further, we report that administration of ribavirin at 50 mg/kg/day starting on days 6, 8, 10, or 12 post-infection resulted in significant protection against HPS in all groups. Administration of ribavirin at 14 days post-infection also provided a significant level of protection against lethal HPS. These data provide *in vivo* evidence supporting the potential use of ribavirin as a post-exposure treatment to prevent HPS after exposure by the respiratory route.

## 1. Introduction

Hantaviruses are enzootic viruses of rodents which cause two severe vascular-leak diseases in humans [1], hemorrhagic fever virus with renal syndrome (HFRS) and hantavirus pulmonary syndrome (HPS). The Old World hantaviruses, which cause HFRS, are harbored by various *Murinae* and *Arvicolinae* rodent species throughout Europe and Asia [2,3]. The second disease, HPS, is caused by New World hantaviruses harbored by *Sigmodontinae* and *Neotominae* rodent species within the Americas. Infections of humans by hantaviruses presumably occur by inhalation of rodent excreta [4,5]. Old (HFRS-causing) and New World (HPS-causing) viruses have a global public health impact estimated at over 100,000 cases each year with lethality ranging from 1% to 50% [1]. In the Americas, HPS affects approximately 300 people a year with a mortality rate of between 15%–50%. There are no FDA-approved vaccines or therapeutic interventions for the prevention or treatment of either HFRS or HPS. 

In human disease, HPS manifests itself in massive pulmonary edema followed by shock and cardiac dysfunction [1,6]. In contrast to other HPS-causing hantaviruses, persuasive evidence suggests person to person dissemination of Andes virus (ANDV) in endemic regions of Chile and Argentina [7,8,9,10,11,12,13]. The Syrian hamster-ANDV model of HPS closely resembles the human disease both in time course and in disease manifestation [14,15,16,17]. Similarities include incubation time, rapid disease onset, infected endothelial cells, pulmonary edema, pleural effusion, and shock. ANDV is lethal in hamster when administered by parenteral (*i.e.*, subcutaneous, intramuscular, or intraperitoneal) or mucosal routes (*i.e*., intranasal, intragastric) [15,18,19]. This model has been used to test candidate countermeasures, such as immune serum, with success [20,21,22,23].

The discovery of new antiviral compounds to prevent hantaviral infection has been limited. Ribavirin (1-β-D-ribofuranosyl-1,2,4-triazole-3-carboxamide) is effective at inhibiting virus replication of HPS and HFRS viruses. Ribavirin is a nucleoside analogue of guanosine. The mechanism of action has been difficult to elucidate because of its ability to interact not only with host cell targets, but also with viral targets. For example, it has been shown that ribavirin can inhibit host-cell inosine monophosphate dehydrogenase as well as Hantaan virus (HTNV) polymerase activity [24], which in turn can increase mutational frequency [25,26,27]. *In vitro*, ribavirin has a 50% effective concentration (EC_50_) value ranging from 1–15 µg/mL when tested in Vero E6 cells with HTNV, ANDV, Sin Nombre virus (SNV), or Maporal virus (MPRLV) [18,28,29,30]. 

In the lethal, suckling mouse model of HFRS with HTNV, ribavirin can prevent mortality when treatment is begun as late as 6 days post-infection [31]. In the infection, suckling mice model with Seoul virus, ribavirin limits virus replication at 25 mg/kg and 50 mg/kg resulting in 68%–81% survival [32,33]. There has been limited research using small animal models of HPS-causing hantaviruses. The deer mouse model of persistent SNV infection shows that ribavirin was effective at inhibiting infection when given at 100 mg/kg in a dose-dependent manner when administered one hour before virus infection [29]. While promising, the deer mouse is a natural rodent reservoir for SNV infection, and does not show any signs of disease. More recently, Safronetz *et al.* showed that treatment with ribavirin up to three days after intraperitoneal challenge with ANDV protected hamsters from lethal HPS [18]. 

Antiviral compound derivatives based upon the structure of ribavirin have shown success at limiting hantaviral replication, for both HFRS and HPS associated hantaviruses, *in vitro* and *in vivo* with lower apparent toxicity [34,35]. Additionally, Favipiravir (T-705, 6-fluoro-3-hydroxy-2-pyrazinecarboxamide), a new broad-spectrum antiviral [36,37], shows an EC_50_ values of 65–93 μM (16–23 μg/mL) against MPRLV, ANDV, SNV and two Old World viruses, Dobrava and Puumala viruses, when tested *in vitro* [30]. T-705 has broad spectrum antiviral activity against other members of the *Bunyaviridae* family [38]. *In vivo* efficacy of T-705 has been reported in the lethal hamster model of ANDV [37].

Clinical trials in China and Korea have shown efficacy in treatment with ribavirin for patients diagnosed with HFRS [39,40]. However, a recent investigation shows that treatment needs to begin before the oliguric phase to prevent mortality [41]. In these clinical studies using intravenous ribavirin treatment of HFRS, human cases caused by HTNV show improvement with a decrease in occurrence of oliguria and severity of renal insufficiency [41]. Human trials using ribavirin for treatment of HPS have been attempted. Unfortunately, due to a limited number of participants the trials have proved inconclusive due to lack of statistical power [42]. Another difficulty with human trials is the fact that most of the study participants were not enrolled in the study until they were already in the cardiopulmonary phase of the disease which usually results in rapid failure and death. This prevents an accurate assessment of the treatment protocol [43,44]. With what little data has been collected the results imply that ribavirin has no effect on disease course outcome. However, no studies have been performed where the patients were in the incubation phase or just prior to cardiopulmonary phase thus allowing treatment protocols the necessary time to prove efficacy.

Here we tested whether ribavirin could be used post-exposure to prevent HPS in an adult animal model of HPS following a respiratory (*i.e*., intranasal) challenge with ANDV. The intranasal (i.n.) route was used to more closely mimic a common route of natural exposure. To confirm that ANDV was indeed sensitive to ribavirin, *in vitro* cell culture assays were run using 10–70 µg/mL of ribavirin. During the process, a quick and effective method of screening for infectivity/replication was developed and is reported herein. Once susceptibility was confirmed, Syrian hamsters were infected i.n. with ANDV and treated with 50, 100 or 200 mg/kg/day of ribavirin, beginning between 1 and 12 days after infection. Efficacy and toxicity were tested simultaneously. These studies show the definite potential ribavirin has as an effective antiviral treatment for HPS. 

## 2. Results and Discussion

Broad spectrum antiviral therapies against the hemorrhagic fever viruses such as the New World hantaviruses is a priority given their high lethality and lack of available FDA-approved treatments [45]. Whether through a natural outbreak or a bioterrorism event, this group of viruses yields little time for medical intervention post-onset, and thus require rapid treatment. Case-fatality for HPS caused by the most prevalent North American and South American hantaviruses, SNV and ANDV, respectively, is 30%–50% even in modern intensive care facilities. This is the second highest mortality associated with hemorrhagic fever viruses. Only the filoviruses exceed these mortality rates in causing mortality up to 90% in some outbreaks in sub-Saharan Africa. Ribavirin has been employed in treatment of several human diseases caused by different hemorrhagic fever viruses including the Old World hantavirus, HTNV [41,46,47], and herein we provide an extensive evaluation of its efficacy in the i.n. challenge model of lethal ANDV infection of adult hamsters.

### 2.1. *In Vitro* Analyses of Ribavirin Inhibition in a Novel Viral Spread Assay and Titer Reduction Assay

**Figure 1:**
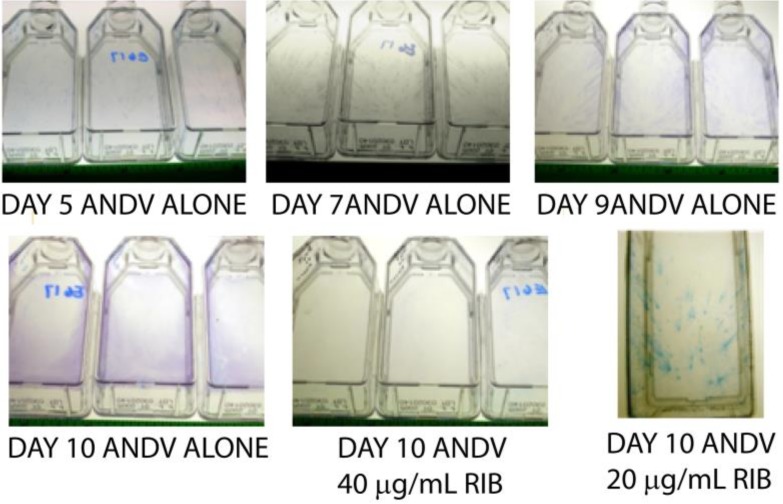
Visual representation of antiviral activity of ribavirin in a comet spread assay with Andes virus (ANDV). Representative culture flasks stained for the presence of the ANDV glycoprotein are shown different days post-infection with ANDV in the presence or absence of ribavirin. ANDV staining on Day 10 at 20 and 40 μg/mL ribavirin shows an inhibition of staining or spread at 40 μg/mL ribavirin but not 20 μg/mL ribavirin. With ANDV alone, an increase in the level of stain is observed over Days 5–10.

We developed a novel viral spread assay to provide a rapid assessment of the potency of ribavirin *in vitro* over a range of concentrations. Vero E6 cells were infected with ANDV virus, allowed to adsorb for 1 h before removing the media and washing two times with PBS. Liquid cell culture media containing from 10–70 µg/mL of ribavirin or media alone was added to the flasks. Cells were fixed from 4 to 10 days after infection and immunostained using an antibody against the glycoprotein. Flasks containing ANDV alone were used for comparison to flasks containing the varying concentrations of ribavirin (Figure 1). In the absence of inhibition, the pattern of immunostaining had a comet-like appearance consisting of an initial focus and then a “tail” of satellite foci presumably resulting from virions released into the media and spread by directional convection currents in the flask. Flasks were scored as either negative (absence of comets), + (few comets), ++ (some comets but less than ANDV alone) or +++ (identical to that of ANDV infected flasks) (Table 1). In the mock-treated cultures, flasks on 4–5 days post-infection showed no viral antigen. On day 5, virus was becoming visible as tiny punctuate spots on the monolayer. By day 7, the virus was visible in the form of comets throughout the cell monolayer. At days 8–10, the viral comets became longer and more numerous until the entire monolayer was completely infected by ANDV (Figure 1). At concentrations of 40 µg/mL and above, ribavirin showed a complete inhibition of virus comets. At 10, 20 and 30 µg/mL, ribavirin showed a delayed appearance of virus comets (days 7–9) suggesting that replication was being repressed but not completely inhibited (Figure 1, Table 1). However, while this assay is novel, it is only qualitative not quantitative so in order to determine the effective inhibitory concentration, a traditional yield reduction assay was also performed.

**Table 1:** Qualitative assessment of antiviral activity in a novel comet assay *.

Concentration	Day 4	Day 5	Day 6	Day 7	Day 8	Day 9	Day 10
ANDV alone	−	+++	+++	+++	+++	+++	+++
10 µg/mL	−	−	−	++	++	++	++
20 µg/mL	−	−	−	+	+	++	++
30 µg/mL	−	−	−	−	−	+	+
40 µg/mL	−	−	−	−	−	−	−
50 µg/mL	−	−	−	−	−	−	−
60 µg/mL	−	−	−	−	−	−	−
70 µg/mL	−	−	−	−	−	−	−

* **−,** no comets formed; +, few comets formed; ++, comets formed not equal to ANDV; +++, comets equal to ANDV alone.

**Figure 2:**
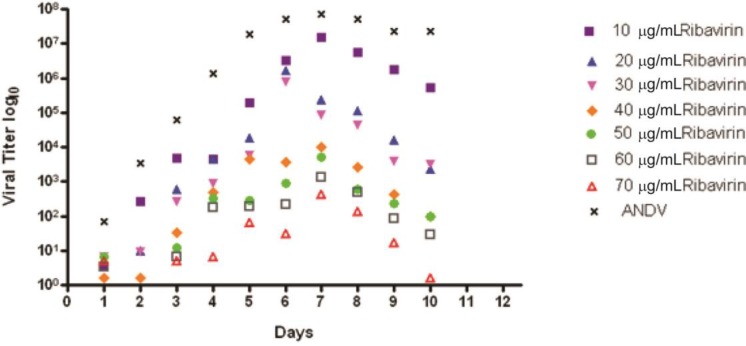
Antiviral activity of ribavirin in a titer reduction assay with ANDV. Vero E6 cells were infected with ANDV and after specific days post-infection viral supernatant was analyzed for viral titer. The viral titers at various concentrations (0–70 μg) of ribavirin are presented.

A titer reduction assay was conducted with supernatant from Vero E6 cells infected with ANDV and treated with varying amounts of ribavirin from 0–70 µg/mL. Two 1 mL aliquots of supernatant were collected daily for 10 days. Aliquots were frozen at −80 °C until a plaque assay could be performed. ANDV alone showed steady growth until maximum growth was reached at 7 days post-infection and then virus output leveled off and remained steady through the rest of the course of the infection (Figure 2). Ribavirin reduced viral titer starting at 10 µg/mL; the 10 µg/mL dose titer levels remained within one log of ANDV over the time course. At doses of 40 µg/mL and higher, ribavirin showed the most significant level of inhibition when compared to ANDV; the difference being a thousand fold or higher (Figure 2). The 50% effective concentration (EC_50_) for inhibition of ANDV on day 5 was 30 μg/mL, which was similar to that observed in the visual comet spread assay.

The viral spread assay presented herein provides a new and effective tool for relatively quick screening of small molecules and biologics. With viruses like the hantaviruses, screening these compounds by traditional methods such as yield reduction is very time consuming, typically taking up to a month to complete. Another positive aspect of this assay is that the absence of comet formation generally correlates to a 1,000 fold reduction in viral titer when compared to yield reduction assays. The data suggests that ANDV was sensitive to ribavirin. The data also indicates that the spread assay while only qualitative does match with the more quantitative titer reduction assay. Thus, the viral spread assay allows candidate antivirals to be quickly screened for efficacy before undergoing the time-consuming yield reduction assay. A caveat is that the relatively large volume of media used in the comet inhibition assay necessitates that orders of magnitude more inhibitor be used to match the concentrations in a 96 or 384 well format.

### 2.2. Ribavirin Efficacy in the Syrian Hamster Model of HPS Following Intranasal Challenge

**Figure 3:**
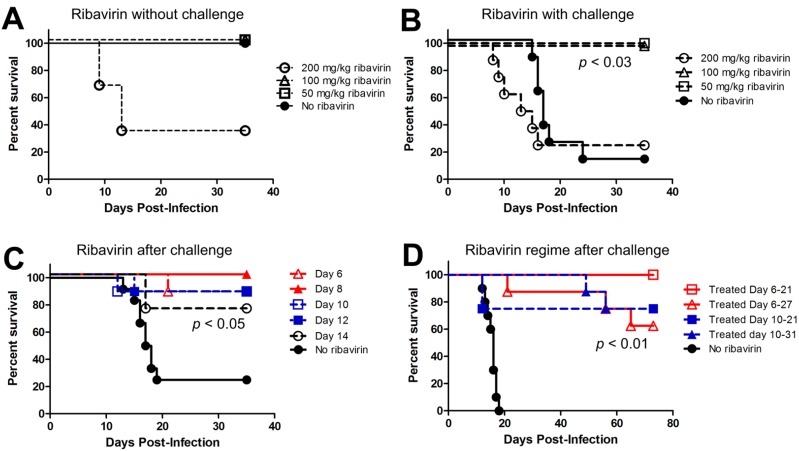
Survival curve of hamsters with mock or ribavirin-treatment following infection with ANDV. (**A**) To test drug toxicity, hamsters were given either 50, 100, or 200 mg/kg/day of ribavirin, or mock treated (no ribavirin), for 21 days and then observed thru 35 days post-infection; (**B**) Hamsters were challenged with 4,000 pfu by the i.n. route and, starting on day 0, given either 50, 100, or 200 mg/kg/day of ribavirin, or mock treated, for 21 days. Animals were observed for signs of disease for 35 days after challenge. Treatment with ribavirin protected animals after infection with ANDV at 100 mg/kg and 50 mg/kg doses (*p* = 0.02 and 0.03, respectively). There was no difference between animals given 200 mg/kg after infection and animals infected without treatment, which confirms the above findings that ribavirin is lethal at a dose of 200 mg/kg; (**C**) Survival curve of hamsters mock or treated with 50 mg/kg/day ribavirin starting at days 6, 8, 10, 12, or 14 following infection with ANDV. All treatment groups had significantly higher survival compared to untreated (*p* < 0.05); (**D**) Animals receiving treatment began at either at day 6 or 10 post-infection survived infection with ANDV for 11, 15 or 21 days. All treatment groups had significantlyhigher survival compared to untreated (*p* < 0.01) Survival analyses were performed by computing pair-wise comparisons to controls using the nonparametric Mantel-Cox test and the resulting *p*-values were adjusted using the Holm-Bonferroni method for multiple comparisons.

In the following experiments, Syrian hamsters were infected i.n. with 4,000 plaque forming units (pfu) with ANDV. The i.n. route of virus challenge was used to more closely mimic the respiratory route of natural exposure. Also, i.n. challenge results in a prolonged incubation period relative to parenteral routes such as intraperitoneal or intramuscular. The mean time-to-death in adult Syrian hamsters after i.n. exposure is 14–26 days depending on the dose [14,20], whereas the intraperitoneal and intramuscular routes result in lethal infections between 1 and 2 weeks. We and others have observed that, regardless of route of exposure, signs of disease are absent until approximately 1–2 days before the animal becomes moribund or succumbs. These signs include dyspnea, tachycardia, lethargy, and occasionally epistaxis [20]. In our initial experiment, groups of hamsters were administered ribavirin at either 50 mg/kg/day, 100 mg/kg/day, 200 mg/kg/day, or mock treated with PBS, starting 1 day post-infection and continued for 21 days. Ribavirin was given twice a day with i.p. injections approximately 10 h apart. Animals were monitored for signs of disease and euthanized when the animals met predetermined criteria (e.g., dyspnea, immobility). To control for drug toxicity, an additional four groups of three uninfected hamsters were treated with 50 mg/kg/day, 100 mg/kg/day, 200 mg/kg/day, or PBS as described above. On day 5, hamsters that were receiving 200 mg/kg/day, both in the infected and uninfected group, started to show signs of illness: weight loss/wasting, diarrhea, and lethargy (Figure 3A,B). Eight animals, 6 infected with ANDV and 2 uninfected, were euthanized due to toxicity caused by ribavirin at this concentration in hamsters. Toxicity of ribavirin in hamsters at 200 mg/kg/day in hamsters has been observed by another group testing ribavirin in hamsters [38]. Uninfected hamsters in either 100 or 50 mg/kg/day groups did not show these signs of drug-induced disease. Untreated hamsters infected with ANDV developed lethal HPS starting on days 12–24 post-infection (mean day-to-death = 17.6 days). None of the animals receiving ribavirin at 50 or 100 mg/kg/day or the uninfected control animals showed signs of illness indicating that ribavirin at 50 or 100 mg/kg/day was capable of preventing death due to HPS after an i.n. challenge (Figure 3B). 

**Figure 4:**
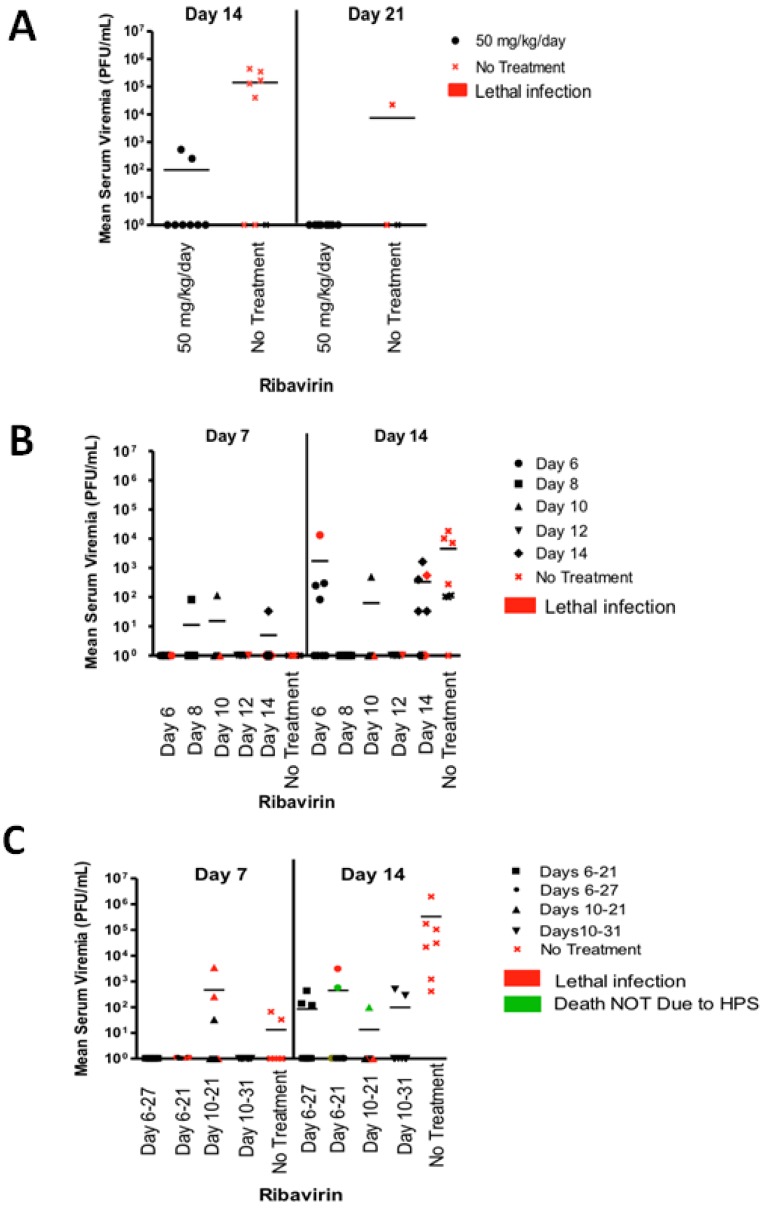
Viremia in ANDV-exposed hamsters treated with ribavirin. Sera collected from ANDV-exposed hamsters from the experiments shown in Figure 3 were evaluated for the presence of infectious ANDV by plaque assay. (**A**) ANDV titers in hamster sera collected 2 or 3 weeks after i.n. challenge with ANDV from the 50 mg/kg/day and no treatment groups are shown. Note that the 100 mg/kg/day sera were all negative for ANDV on all time points (data not shown); (**B**) ANDV titers in hamster sera collected 1 or 2 weeks after i.n. challenge with ANDV. Ribavirin treatment was started on days 6, 8, 10, 12, or 14 days after challenge; (**C**) ANDV titers in hamster sera collected 1 or 2 weeks after i.n. challenge with ANDV. Ribavirin treatment started on day 6 or 10 and continued for 11, 15, or 21 days. Red symbols represent hamsters that ultimately developed lethal infections. Green symbols indicate animals that succumbed after anesthesia or for other causes other than HPS. Lines indicate mean ANDV titers for each group.

**Figure 5:**
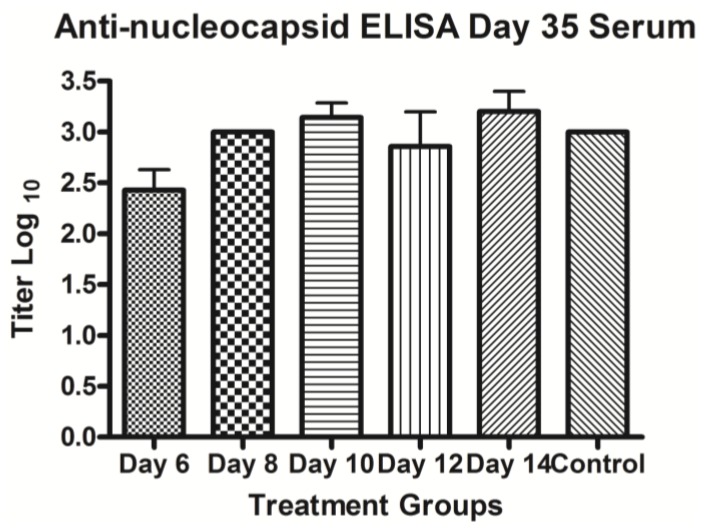
ELISA titers on day 35 post-ANDV infection of hamster. Titer (y-axis) is shown on Day 35 in sera collected from measurement by ELISA with antibody to the nucleocapsid protein. Various treatment and control (no treatment) groups (X-axis) are shown.

Analysis of serum samples collected after challenge demonstrated that ribavirin treatment starting 1 day after challenge reduced or completely abolished the presence of live virus. No virus was detected 7 days after challenge in all groups (data not shown). By 14 days after infection, the No Treatment and 50 mg/kg/day ribavirin groups showed the presence of virus in the sera (Figure 4A). Virus was not detected in the sera of the 100 or 200 mg/kg/day treatment groups at any time after challenge (data not shown). By 21 days, all of the surviving animals except one in the No Treatment group had cleared the viremia. These results suggested the higher concentrations of ribavirin completely inhibited ANDV replication *in vivo*. Animals treated with 50 mg/kg/day were only partially protected from virus replication. To ensure that those animals that survived did so because of repression of virus replication, and not because of a lack of virus exposure, ELISA’s were performed to measure anti-ANDV nucleocapsid antibody levels. All animals which were exposed to virus were positive for anti-nucleocapsid IgG antibody by enzyme-linked immunosorbent assay (ELISA), whereas all day-7 prebleeds were negative, indicating that all the animals were indeed exposed to ANDV (Figure 5). All animals that were not exposed to ANDV did not show the presence of any viral titer or any evidence of seroconversion indicating that cross contamination of cages in the study room did not occur. 

### 2.3. Last Effective Day Post-Exposure for Treating HPS

Since a dose of 50 mg/kg/day was effective at preventing HPS, we asked if this dose would be effective if begun later after ANDV challenge. Groups of eight hamsters were challenged with ANDV as described above. Ribavirin treatment was started at 6, 8, 10, 12 or 14 days after challenge. Ribavirin was given as previously described, two times a day approximately 10 h apart, and given for a full 21 days. Animals that were not treated with ribavirin were given PBS on the same schedule as the treated groups (Figure 3C). Untreated animals became ill and were euthanized starting as early as day 12. Three animals not receiving ribavirin survived (75% lethal) indicating the challenge was not as lethal in previous experiments (88% lethal). While lethal infection occurred in all groups except for those animals receiving treatment on day 8, treatment starting as late as day 12 was effective (*p* < 0.05) at preventing HPS. Treatment starting on day 14 prevented death in 75% of the animals (*p* = 0.034). 

Seven days post-challenge, the majority of animals in all treatment groups were negative for infectious ANDV (Figure 4B). Fourteen days post challenge, approximately half of the animals in the Day 6, 14, and No Treatment groups showed signs of viremia. Twenty-one days after challenge, all of the surviving hamsters were negative for the presence of infectious ANDV (data not shown). This suggests that suppression of virus replication after infection can prevent HPS and allow the animals to clear infectious virus. Serum from 35 days post-infection, but not the day-7 prebleeds, showed the presence of anti-ANDV nucleocapsid antibodies in all groups, treated and untreated, suggesting that ANDV infection occurred in all exposed hamsters (Figure 5). Thus, ribavirin at 50 mg/kg/day administered as late as 12 days after i.n. exposure is capable of preventing HPS in the hamster model. Day 12 is just 5 days before the mean time-to-death, 17.6 days. The onset of clinical signs is generally 1–2 days before the mean day-to-death.

### 2.4. Confirming That Ribavirin can Be Used to Prevent HPS even When Treatment Starts 6–10 Days after a Respiratory Exposure

To confirm that late administration of ribavirin could confer protection against an i.n. challenge, and to test the possibility that the duration of treatment could be reduced; groups of eight to ten hamsters were infected i.n. with 4,000 pfu of ANDV. Ribavirin was administered at 50 mg/kg/day as before between days 6–21 (15 days), 6–27 (21 days) or between 10–21 (11 days) and 10–31 (21 days) days post-infection. Severe disease (requiring euthanasia) was first observed on day 12 and continued thru day 17 with the mean-time-to-death being 15.4 days. Two animals in the Day 10 group (10–21) developed HPS on day 12 with the remaining animals showing no signs of illness. All animals receiving treatment between Days 6–21 and most of the animals treated between Days 6–27 remained healthy and did not develop HPS (Figure 3D). To look for evidence of long-term sequelae [48], this experiment was continued for 10 weeks. Unexpectedly, two hamsters in the Days 6–21 group and two in the Days 10–21 group succumbed to illness beginning at 49. Necropsies revealed liver necrosis with signs of regeneration (data not shown). Viral antigen was not found within the liver tissue as measured by immunohistochemistry (data not shown), suggesting that the hamsters died because of liver failure of unknown etiology, and not hantavirus infection. This experiment confirmed that ribavirin treatment starting as late as day 10 post exposure, and administered for as few as 11 days, could prevent mortality due to HPS (survival analysis compared to untreated, *p* < 0.01). Due to the toxicities of this drug, further investigations to determine the minimal duration of ribavirin treatment to prevent HPS are warranted. 

Untreated animals and animals receiving ribavirin between days 10–21, that died around day 12 due to HPS (Figure 3D), had detectable viral titers on day 7 (Figure 4C). By day 14, a fraction of animals all groups had detectable levels of ANDV in the serum as measured by plaque assay. By day 21 there was no detectable infectious virus in any of the treated animals (data not shown). On day 35 and day 73, which was the end point of the experiment, none of the ribavirin treated animals showed the presence of virus (data not shown). As observed in earlier experiments, serum from 35 days post-infection, but not the day-7 prebleeds, showed the presence of anti-ANDV nucleocapsid antibodies in all groups, treated and untreated, suggesting that ANDV infection occurred in all exposed hamsters (Figure 5). 

## 3. Experimental

### 3.1. Viruses and Cells

ANDV strain Chile-917869 [14] was propagated in Vero E6 cells (Vero C1008; ATCC CRL 1586). Cells were maintained in Eagle’s minimum essential medium with Earle’s salts (EMEM) containing 10% fetal bovine serum (FBS), 10 mM HEPES, pH 7.4, and antibiotics (penicillin [100 U/mL], streptomycin [100 µg/mL], gentamicin sulfate [50 µg/mL]) in a 5% CO_2_ incubator at 37 °C. ANDV passage 2, twice-plaque-purified virus stock preparation, was previously described [14].

### 3.2. Virus Yield Reduction Assay

The antiviral activity of ribavirin was evaluated by a plaque reduction assay in Vero E6 cells infected with ANDV strain Chile-917869. Six-seven day old confluent Vero E6 cells grown in T150 flasks were infected with ANDV at a multiplicity of infection of 0.1 followed by absorption for 1 h. After removing virally infected media and washing the cells in PBS, the media was replaced with complete EMEM (cMEM) containing ribavirin (ICN Pharmaceuticals, Costa Mesa, CA, USA) at concentrations ranging from 0–70 µg/mL and the cells were incubated at 37 °C and 5% CO_2_. Every day 2 mL of supernatant were harvested from each flask and frozen at −80 °C until they were subjected to plaque assay to measure progeny virus released into the supernatant.

### 3.3. Serum Plaque Assay

Hantaviral plaque assays to measure infectious virus in sera were performed as previously described [14]. Briefly, serum samples were centrifuged at 10,000 ×*g* for 10 s to pellet any debris before dilution. Supernatants were used to prepare 1:10 dilutions. Once sample dilutions were made, 200 µL was added to each well of a 6-well Costar plate containing 7 day old Vero E6 cells. After a 1 h adsorption at 37 °C and 5% CO_2_, 3 mL overlay medium was added to each well as previously described [14]. Plates were incubated at 37 °C and 5% CO_2_ for 7 days at which point they were stained with a 2 mL/well overlay medium containing 5% fetal bovine serum and 5% neutral red solution (Invitrogen, Carlsbad, CA, USA). Plates were incubated for an additional 3 days at 37 °C or until plaques were visible and countable.

### 3.4. Viral Spread (Comet) Assay

The antiviral activity of ribavirin was evaluated by a virus spread inhibition assay in Vero E6 cells infected with ANDV strain Chile-917869. One week-old confluent Vero E6 cells grown in T25 flasks were infected with ANDV at 15 µL of a 3.6 × 107 pfu/mL stock in 10 mL followed by adsorption for 1 h. An additional 10 mL of cMEM media was added to the flask at the end of the hour incubation period. The flasks were placed in a 37 °C and 5% CO_2_ incubator with the plate containing the flasks set at a slight angle, which influenced the direction of the comet-like immunostaining pattern. Flasks were fixed in 2 mL of a 50% methanol and acetone mixture for 10 min and washed with 1 mL of PBS twice. Cells were immunostained for the presence of viral antigen using antibody mAb-3d7, which is directed toward the G_c_ glycoprotein. After removal of the PBS, 1 mL of the primary antibody solution (3% fetal bovine serum and a 1:500 dilution of mAb-3d7 in PBS) was added to the flasks which were then placed on a rocker at room temperature for 2 h. The antibody mixture was subsequently removed from the flasks and the monolayer was washed with 2 mL of PBS 2 times. One mL of the secondary antibody mixture (3% fetal bovine serum, a 1:1,000 dilution of HPO-Bethyl G and M IgG1 and PBS) was added to the flasks and allowed to rock at room temperature for one and one half hours. The secondary antibody mixture was then removed from the flasks and the flasks were washed with 2 mL of PBS 2 times. One mL of substrate solution, KPL-True Blue Peroxidase Substrate, (KPL, Kirkegaard and Perry Laboratories Inc., Gaithersburg, MD, USA) was added to each flask and allowed to develop for 10 min. The substrate solution was removed and washed with 2 mL of distilled water. After removal of the water, flasks were air-dried and examined for the presence of virally stained cells. Viral inhibition was determined by comparing uninfected flasks with flasks containing antiviral compounds. Compounds that were inhibitory did not show the presence of viral “comets”—showing spread of the ANDV from cell to cell. 

### 3.5. Intranasal Infection of Hamsters with ANDV

Syrian female hamsters, 6–8 week old, were obtained from Harlan (Indianapolis, IN, USA) and were used for all animal studies. Hamsters were routinely used within 1–2 weeks after shipment from the vendor. Hamsters younger than 7 weeks at time of challenge were not used in this study because we, and others, have observed a shorter incubation time (*i.e*., 10–13 days *versus* 14–26 days) following i.n. challenge in the younger hamsters (unpublished data and [18,19]). Hamsters were identified by an ear-tag. Hamsters were bled by vena cava at day 7 prior to infection; 0.5 mL of blood was taken. Hamsters were moved into the biosafety level 4 laboratory (BSL-4) to acclimate at least 3 days prior to infection. Animals were anesthetized by isoflurane and infected i.n. with 4,000 pfu of ANDV in 50 µL of PBS delivered as 25 µL per naris. Control animals were given 50 µL of PBS without ANDV. Hamsters were injected with 0.1 mL/100 g of body weight with ketamine-acepromazine-xylazine mixture before bleeds (0.5 mL) on 7, 14, 21, and 35 days post-infection. Starting one day post-infection hamsters were given 200 mg/kg/daily, 100 mg/kg /daily or 50 mg/kg/daily of ribavirin dissolved in PBS and filter sterilized as two injections in a volume of 0.5 mL approximately 10 h apart. Hamsters receiving no treatment were injected with filtered PBS in the same volume and according to the same dosing schedule as the treated hamsters. Hamsters were monitored two to three times daily. All work involving ANDV infected hamsters was performed in a BSL-4. Research was conducted in compliance with the animal welfare act and other federal statutes and regulations relating to animals and experiments involving animals adhered to the principles stated in the Guide for the Care and Use of Laboratory Animals, National Research Council, 2011. The facility where this research was conducted is fully accredited by the Association for Assessment and Accreditation of Laboratory Animal Care International.

### 3.6. Post-Exposure Prophylaxis for Treating HPS

Post-exposure prophylaxis hamsters were given injections of ribavirin 50 mg/kg/daily divided into two equal injections (10 h apart) beginning at 6, 8, 10, 12, or 14 after challenge and continuing for 21 days. Animals that were not treated with ribavirin were given PBS on the same schedule as the treated groups. Groups of eight hamsters were challenged with ANDV as described above. Hamsters were observed for 35 days at which point survivors were euthanized. The second iteration of post-exposure prophylaxis hamsters were given injections of ribavirin 50 mg/kg/daily divided into two equal injections continuing for either 21 days or up until day 21. Hamsters were observed for 73 days at which point survivors were euthanized. Research was conducted in compliance as stated above in Section 3.5.

### 3.7. ELISA

Antibodies to ANDV nucleocapsid cross-react with Puumala virus nucleocapsid. This cross-reactivity makes it possible to use a Puumala virus nucleocapsid-based ELISA to detect antibodies to the ANDV nucleocapsid. Anti-nucleocapsid ELISAs were performed as described previously [14,49]. The plasmid pPUUSXdelta (kindly provided by F. Elgh) was expressed in *Escherichia coli* BL21 (DE3) cells (Novagen, Madison, WI, USA) to generate histidine-tagged truncated Puumala virus nucleocapsid protein. The protein was affinity purified using a Ni-nitrilotriacetic acid column (Qiagen, Valencia, CA, USA). Hamster sera was gamma irradiated on dry ice (3 million rad from a ^60^C source) and heat inactivated before being tested in ELISA.

### 3.8. Statistical Analyses

Survival analyses were performed by computing pair-wise comparisons to controls using the nonparametric Mantel-Cox test using the program R (version 2.13.0). The resulting *p*-values are reported after *a posteriori* adjustments using the Holm-Bonferroni method for multiple comparisons.

## 4. Conclusions

Clinical studies have shown that, if begun early in the disease process, ribavirin can be effective against hantaviruses causing HFRS [39,41]. Clinical trials for ribavirin for HPS have been limited and inconclusive [42,43]. The efficacy of ribavirin in treatment of HPS in hamsters will hopefully provide the incentive to open large clinical studies across borders throughout the Americas. This research provides further evidence that this drug can be effectively used in treating this rapid and life-threatening disease if given before onset of symptoms. Here we have shown significant efficacy if treatment began as late as 14 days after exposure, only days before signs of disease. Ribavirin has known side effects, such as hemolytic anemia and potential teratogenicity, which make therapy difficult and potentially dangerous [45,50]. The shorter the period of time those patients remain on treatment, the less chance that they will experience detrimental side effects. The use of an antiviral therapeutic starting on day 10 and administered for only 11 days in the hamster-ANDV model without observing viral re-emergence is a remarkable finding. This suggests that the immune system is capable of clearing the virus if replication can be inhibited before high levels of viremia occur. The ANDV/hamster i.n. challenge studies reported herein, combined with ANDV/hamster i.p. challenge studies reported by others [18], provide health care workers and scientists information that can be used for decision making as to whether or not ribavirin will be of clinical benefit following a needle stick, animal bite, or other event with a high probability of hantavirus exposure. Future research aims to identify noninvasive methods to detect early signs of disease in the hamster model of HPS. Such methods would allow for more accurate trigger-to-treat times for studies of candidate therapeutics.
